# Author Correction: The cerebellum contributes to generalized seizures by altering activity in the ventral posteromedial nucleus

**DOI:** 10.1038/s42003-024-05885-4

**Published:** 2024-02-16

**Authors:** Jaclyn Beckinghausen, Joshua Ortiz-Guzman, Tao Lin, Benjamin Bachman, Luis E. Salazar Leon, Yu Liu, Detlef H. Heck, Benjamin R. Arenkiel, Roy V. Sillitoe

**Affiliations:** 1 Department of Pathology and Immunology, Baylor College of Medicine, Houston, TX USA; 2 Department of Neuroscience, Baylor College of Medicine, Houston, TX USA; 3 Jan and Dan Duncan Neurological Research Institute of Texas Children’s Hospital, 1250 Moursund Street, Suite 1325 Houston, TX USA; 4 Program in Developmental Biology, Baylor College of Medicine, Houston, TX USA; 5 Department of Molecular and Human Genetics, Baylor College of Medicine, Houston, TX USA; 6 Department of Biomedical Sciences, University of Minnesota Medical School, Duluth, 103515 University Dr., Duluth, MN USA

**Keywords:** Neural circuits, Epilepsy

Correction to: *Communications Biology* 10.1038/s42003-023-05100-w, published online 15 July 2023

The cited scientist’s name should be Irving S. Cooper instead of Edward Cooper. The original article has been corrected.

