# Supplementary material for: The cerebellum contributes to generalized seizures by altering activity in the ventral posteromedial nucleus
Source: Commun Biol. 2023 Jul 15;6:731. doi: 10.1038/s42003-023-05100-w (PMC10349834; doi:10.1038/s42003-023-05100-w)
Supplement: Supplementary file 2 — Supplementary Information [file 42003_2023_5100_MOESM2_ESM.pdf]

SUPPLEMENTARY INFORMATION

Supplementary Figure 1

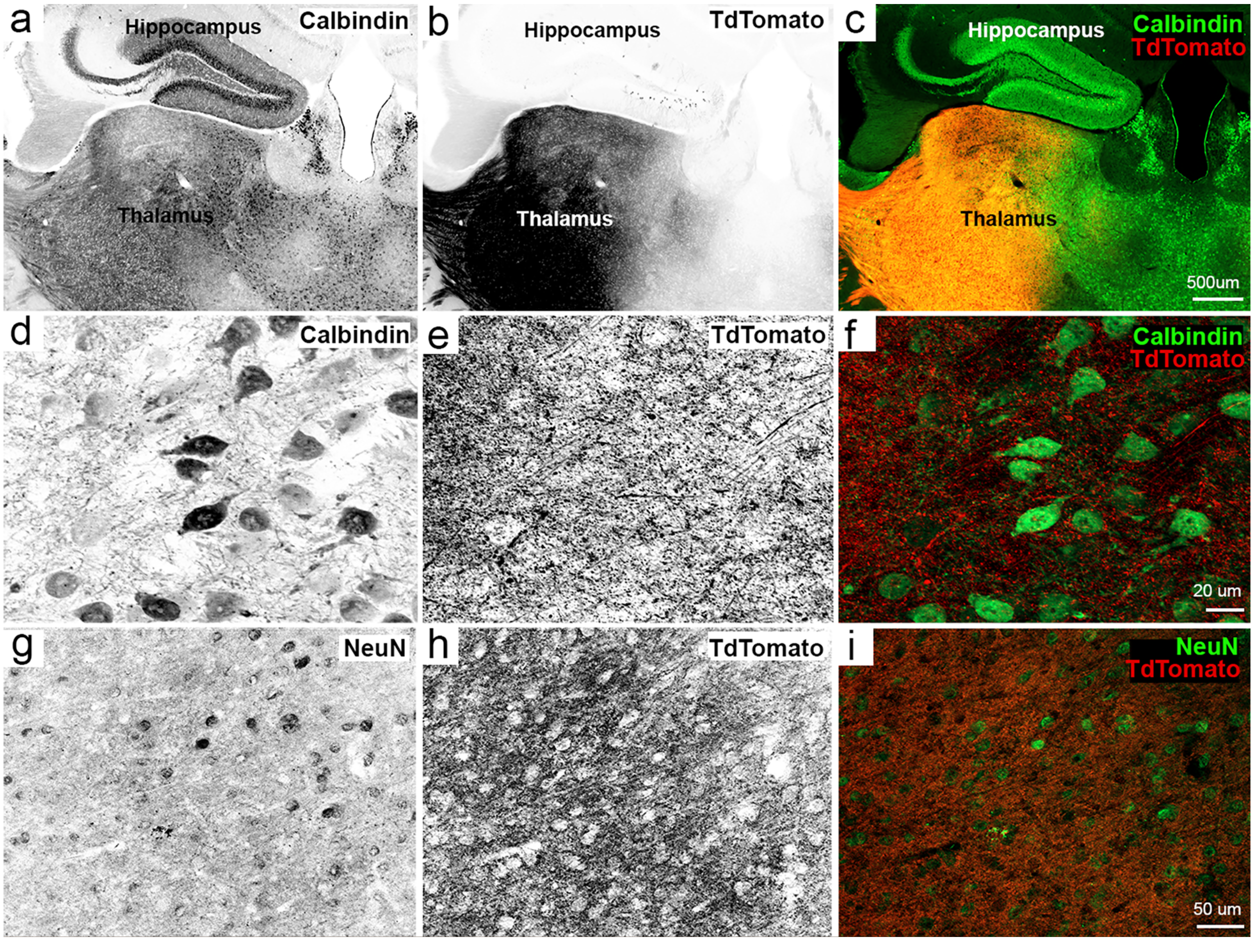

Supplementary Figure 1: Ntsr1^{Cre} *is not expressed in cell bodies of thalamic neurons*. (a-f) Serially magnified images of calbindin staining in the thalamus, which is differentially expressed in various thalamic nuclei, demonstrates no colocalization between TdTomato (red) and calbindin-positive (green) thalamic neurons. Scale bars are 500µm (c) and 20µm (f). (g-i) Staining with a pan neuronal marker, NeuN, does not colocalize with TdTomato signal in the thalamus. Instead, *Ntsr1*^{Cre} is expressed in the space around resident thalamic cell bodies (h) as local axons or fibers of passage originating from other *Cre*-positive brain regions. Scale bar is 50um (i).

Supplementary Figure 2

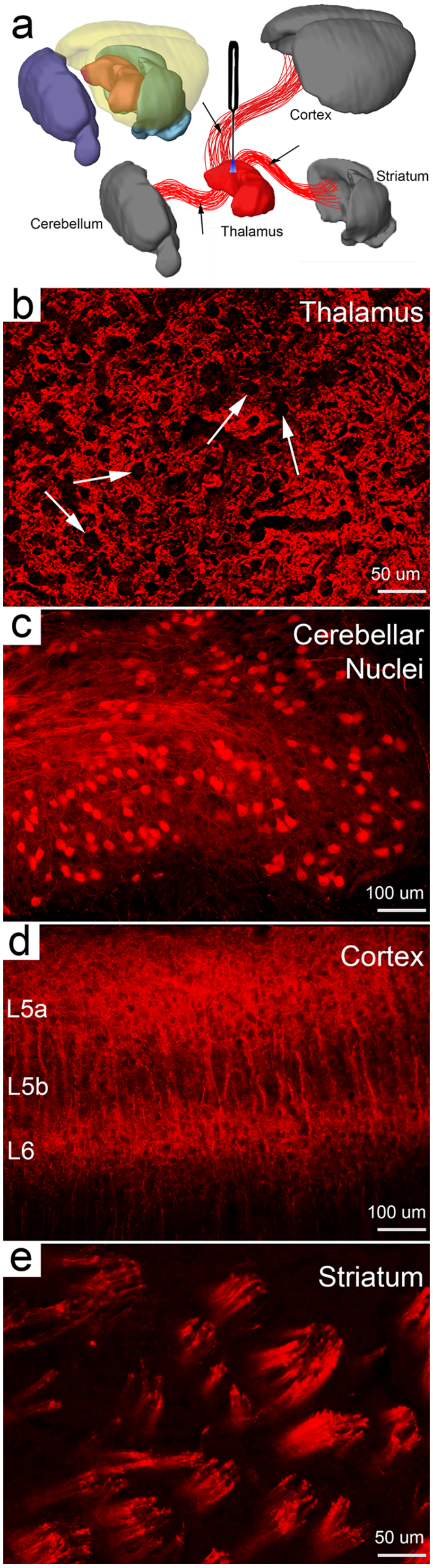

Supplementary Figure 2: Optogenetic modulation of thalamus instead targets input from extra-thalamic regions. (a) Because the local thalamic neurons themselves do not express *Cre* in $Ntsr1^{Cre}$ animals, manipulation of their activity is indirect by modulating *Cre*-expressing axons and fibers of passage from regions outside the thalamus itself. Images of brain regions adapted and modified from BrainExplorer2, Allen Brain Atlas. (b-e) High power images of *Cre*-induced reporter expression patterns in the thalamus (scale bar = 50μm) (b), cerebellar nuclei (scale bar = 100μm) (c), cortex (scale bar = 100μm) (d), and striatum (scale bar = 50μm) (e). Note the distinction of the expression pattern of the thalamus compared (b) to other regions (c-e), where fibers but not the resident neurons have expression. These areas are depicted by absence of localized fluorescence (arrows).

Supplementary Figure 3

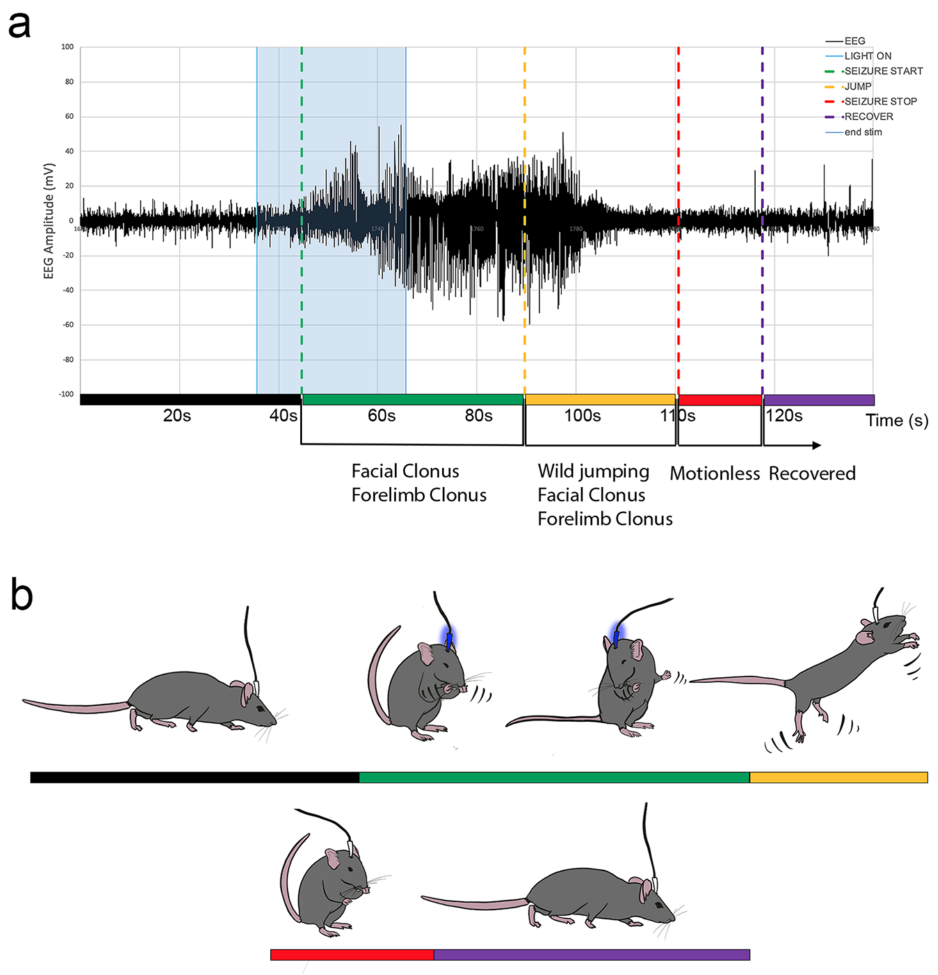

Supplementary Figure 3: Mice exhibit a reproducible series of seizure behaviors during optogenetic stimulation of the VPM. (a) An EcoG trace of the ipsilateral motor cortex is labeled by corresponding behaviors marked by color-coded dashed lines. (b) The corresponding mouse behaviors are schematized as they fall within the EcoG trace, as denoted by colored bars on both (a) and (b).

Supplementary Figure 4

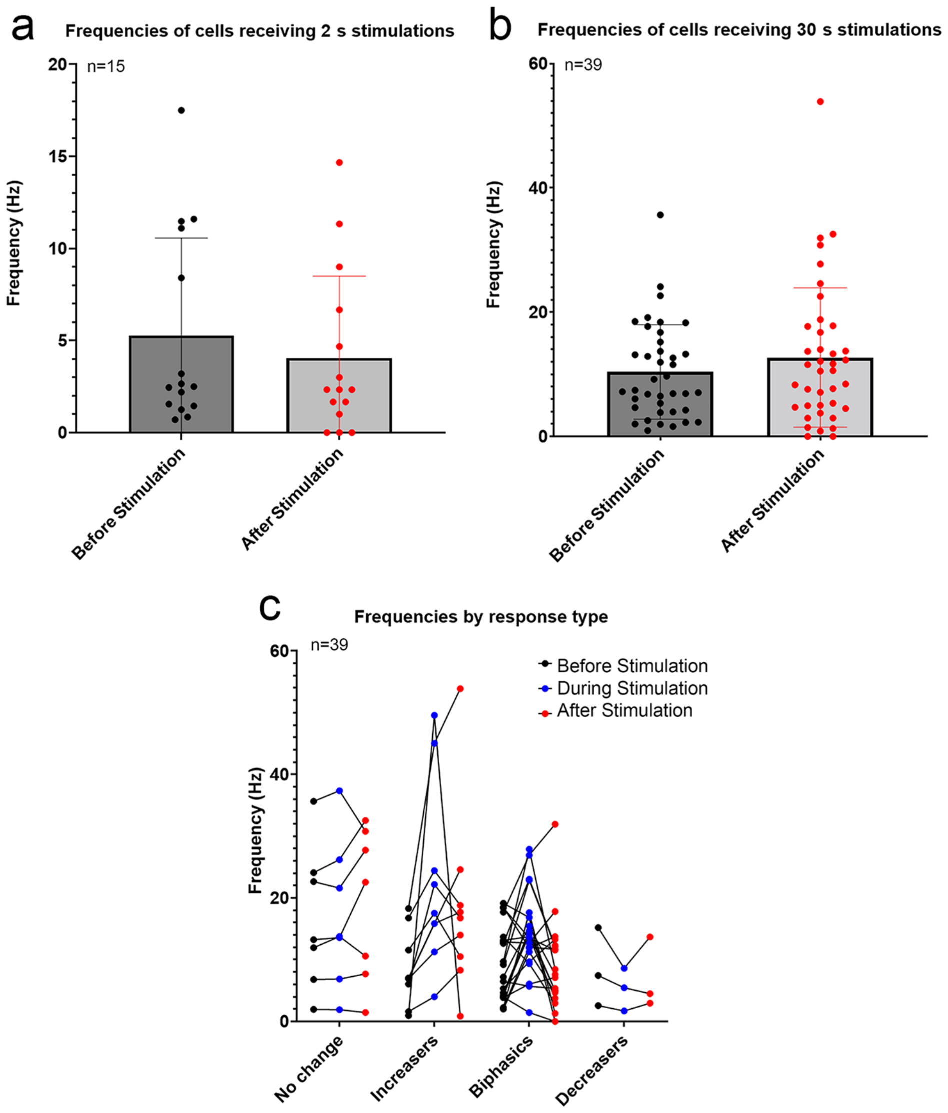

Supplementary Figure 4: Quantification of single cell thalamic frequencies before and after stimulation reveals rapid return to baseline. (a) Scatter plot of before (20 seconds analyzed) versus after (20 seconds analyzed) frequencies of cells receiving 2-second, subthreshold light stimulations demonstrating no statistical significance ($p = 0.1479$, $n = 15$ cells). (b) Plot of before (20 seconds analyzed) versus after (20 seconds analyzed) frequencies of only those cells receiving 30-second light stimulations again did not demonstrate statistical significance ($p = 0.1100$, $n = 39$ cells). (c) Single cell responses before (black), during (blue), and after (red) (20 seconds, 30 seconds, and 20 seconds, respectively) calculated as the average frequency

demonstrates that baseline (before, black) activity was not a significant indicator for cellular response ($p > 0.05$, $n = 39$ cells). Error bars in all graphs represent SEM.
