# Supplementary material for: The cerebellum contributes to generalized seizures by altering activity in the ventral posteromedial nucleus
Source: Commun Biol. 2023 Jul 15;6:731. doi: 10.1038/s42003-023-05100-w (PMC10349834; doi:10.1038/s42003-023-05100-w)
Supplement: Supplementary file 3 — Description of Additional Supplementary Files [file 42003_2023_5100_MOESM3_ESM.pdf]

Description of Additional Supplementary Files

File Name: Supplementary Data 1
Description: Source data for all plots

File Name: Supplementary Video 1
Description: Optogenetic delivery of 30Hz light to the VPM elicits tonic-clonic seizures. Video representation of a stage 6 seizure with a 30 second light stimulation to the VPM showing examples of the progression from facial and forelimb clonus to loss of posture in a mouse exhibiting a light-induced seizure. The "During Stimulation" segment occurs 6 seconds after the start of the light pulse. "Stage 6 seizure continues after light pulse" as shown in the video beginning 2.5 seconds after the end of the 30 second light stimulation period. The last segment in which the mouse is completely recovered was taken 1 minute after the end of the 30 second light stimulation period. All mice, including those with severe phenotypes, experience a full recovery of general behavior after the seizures resolve.

File Name: Supplementary Video 2
Description: Optogenetic seizure induction is specific to the thalamic VPM. Stimulation of surrounding thalamic nuclei, all Cre-expressing, does not elicit any overt behavioral phenotype. In this example, we show the effects of 30-Hz light delivery to the ventral posterolateral nucleus (VPL), which receives some common input with the VPM and is directly adjacent to the seizure-inducing region.

File Name: Supplementary Video 3
Description: Behavioral similarities between KA-induced and optogenetic-induced seizures. The behavioral phenotype of KA induced seizures and the optogenetic model are identical. Both exhibit forelimb and facial clonus, Straub tail, and loss of body posture.

File Name: Supplementary Video 4
Description: Pharmacological silencing of the cerebellar nuclei eliminates the ability to induce seizures. Before administration of lidocaine, the mouse exhibits a severe motor seizure upon light delivery to the VPM. After bilateral lidocaine delivery to the interposed cerebellar nuclei, light stimulation does not evoke any overt behavioral changes. However, after lidocaine washout, the seizure can once again can be elicited by light stimulation directed into the VPM.
