# Supplementary material for: The cerebellum contributes to generalized seizures by altering activity in the ventral posteromedial nucleus
Source: Commun Biol. 2023 Jul 15;6:731. doi: 10.1038/s42003-023-05100-w (PMC10349834; doi:10.1038/s42003-023-05100-w)
Supplement: Supplementary file 10 — Reporting Summary [file 42003_2023_5100_MOESM10_ESM.pdf]

nature portfolio

Corresponding author(s): Sillitoe, Roy

Last updated by author(s): 06/15/2023

Reporting Summary

Nature Portfolio wishes to improve the reproducibility of the work that we publish. This form provides structure for consistency and transparency in reporting. For further information on Nature Portfolio policies, see our Editorial Policies and the Editorial Policy Checklist.

Statistics

For all statistical analyses, confirm that the following items are present in the figure legend, table legend, main text, or Methods section.

n/a	Confirmed	
☐	☒	The exact sample size (*n*) for each experimental group/condition, given as a discrete number and unit of measurement
☐	☒	A statement on whether measurements were taken from distinct samples or whether the same sample was measured repeatedly
☐	☒	The statistical test(s) used AND whether they are one- or two-sided<br>*Only common tests should be described solely by name; describe more complex techniques in the Methods section.*
☐	☒	A description of all covariates tested
☐	☒	A description of any assumptions or corrections, such as tests of normality and adjustment for multiple comparisons
☐	☒	A full description of the statistical parameters including central tendency (e.g. means) or other basic estimates (e.g. regression coefficient) AND variation (e.g. standard deviation) or associated estimates of uncertainty (e.g. confidence intervals)
☐	☒	For null hypothesis testing, the test statistic (e.g. *F*, *t*, *r*) with confidence intervals, effect sizes, degrees of freedom and *P* value noted<br>*Give P values as exact values whenever suitable.*
☒	☐	For Bayesian analysis, information on the choice of priors and Markov chain Monte Carlo settings
☐	☒	For hierarchical and complex designs, identification of the appropriate level for tests and full reporting of outcomes
☐	☒	Estimates of effect sizes (e.g. Cohen's *d*, Pearson's *r*), indicating how they were calculated

Our web collection on statistics for biologists contains articles on many of the points above.

Software and code

Policy information about availability of computer code

Data collection	All EEG was recorded through Spike2 software and delivered using a CED Power1401 data acquisition interface (CED, Cambridge, UK).
Data analysis	EEG and single cell quantifications were analyzed in Spike2 2019 or MatLab. Code available upon request.

For manuscripts utilizing custom algorithms or software that are central to the research but not yet described in published literature, software must be made available to editors and reviewers. We strongly encourage code deposition in a community repository (e.g. GitHub). See the Nature Portfolio guidelines for submitting code & software for further information.

Data

Policy information about availability of data

All manuscripts must include a data availability statement. This statement should provide the following information, where applicable:

- Accession codes, unique identifiers, or web links for publicly available datasets
- A description of any restrictions on data availability
- For clinical datasets or third party data, please ensure that the statement adheres to our policy

Data generated in the experiments presented in the current study are available from the corresponding author upon request.

Human research participants

Policy information about studies involving human research participants and Sex and Gender in Research.

Reporting on sex and gender	*Use the terms sex (biological attribute) and gender (shaped by social and cultural circumstances) carefully in order to avoid confusing both terms. Indicate if findings apply to only one sex or gender; describe whether sex and gender were considered in study design whether sex and/or gender was determined based on self-reporting or assigned and methods used. Provide in the source data disaggregated sex and gender data where this information has been collected, and consent has been obtained for sharing of individual-level data; provide overall numbers in this Reporting Summary. Please state if this information has not been collected. Report sex- and gender-based analyses where performed, justify reasons for lack of sex- and gender-based analysis.*
Population characteristics	*Describe the covariate-relevant population characteristics of the human research participants (e.g. age, genotypic information, past and current diagnosis and treatment categories). If you filled out the behavioural & social sciences study design questions and have nothing to add here, write "See above."*
Recruitment	*Describe how participants were recruited. Outline any potential self-selection bias or other biases that may be present and how these are likely to impact results.*
Ethics oversight	*Identify the organization(s) that approved the study protocol.*

Note that full information on the approval of the study protocol must also be provided in the manuscript.

Field-specific reporting

Please select the one below that is the best fit for your research. If you are not sure, read the appropriate sections before making your selection.

☒ Life sciences ☐ Behavioural & social sciences ☐ Ecological, evolutionary & environmental sciences

For a reference copy of the document with all sections, see nature.com/documents/nr-reporting-summary-flat.pdf

Life sciences study design

All studies must disclose on these points even when the disclosure is negative.

Sample size	Sample size was not determined using a priori power analysis but was based on the criteria for significance in observation and based on those used in previous similar publications (White et al, 2017).
Data exclusions	Data was not excluded unless otherwise explicitly stated in the text.
Replication	We conducted our experiments multiple times to ensure that our findings were consistent across multiple runs. Additionally, we used multiple replicates for each experimental condition to increase the reliability of our results.<br><br>Furthermore, we utilized standardized protocols and procedures for data collection and analysis to ensure that our methods were consistent and repeatable. We also made efforts to document our procedures and data analysis in detail, including recording any deviations or modifications to our methods. We also conducted blind experiments to minimize biases that could impact the reproducibility of our findings.
Randomization	Animals were assigned to experimental groups based on genotype, and littermates without ChR2 expression were used as controls. As such, there was no bias in group assignments.
Blinding	Blinding was utilized for the duration of each experiment - animals were assigned masked numbers with a "key" identifying their actual genotype. Surgeries and subsequent behavior or additional experiments were performed without knowing the genotype of each animal. The subsequent analyses were also approached in a blind fashion, as the names of each animals' dataset was obscured until analysis was completed.

Reporting for specific materials, systems and methods

We require information from authors about some types of materials, experimental systems and methods used in many studies. Here, indicate whether each material, system or method listed is relevant to your study. If you are not sure if a list item applies to your research, read the appropriate section before selecting a response.

Materials & experimental systems

n/a	Involved in the study
☐	☒ Antibodies
☐	☒ Eukaryotic cell lines
☒	☐ Palaeontology and archaeology
☐	☒ Animals and other organisms
☒	☐ Clinical data
☒	☐ Dual use research of concern

Methods

n/a	Involved in the study
☒	☐ ChIP-seq
☒	☐ Flow cytometry
☒	☐ MRI-based neuroimaging

Antibodies

Antibodies used

Antibodies used for immunohistochemistry (primary antibodies) were: calbindin (Rb and mouse (Ms), Swant, 1:10,000); NeuN (Rb, Millipore, 1:500); NFH (Ms, Covance, 1:1,500); TH (Rb, Millipore, 1:500); GFP (Ch, Abcam, 1:1,000); Secondary antibodies were: donkey anti-mouse, anti-rabbit or anti-guinea pig secondary antibodies conjugated to Alexa 488, 555 or 647 fluorophores (Invitrogen), all diluted to 1:1,500

Validation

We also verified the specificity of each primary antibody by conducting appropriate controls, including positive and negative controls, and testing the antibody on samples known to express the target protein. The antibody profiles for each primary antibody were also cross-checked in online databases, such as the Antibody Registry and Antibodypedia, to confirm their specificity and to assess any potential cross-reactivity with other proteins.

Eukaryotic cell lines

Policy information about cell lines and Sex and Gender in Research

Cell line source(s)

State the source of each cell line used and the sex of all primary cell lines and cells derived from human participants or vertebrate models.

Authentication

Describe the authentication procedures for each cell line used OR declare that none of the cell lines used were authenticated.

Mycoplasma contamination

Confirm that all cell lines tested negative for mycoplasma contamination OR describe the results of the testing for mycoplasma contamination OR declare that the cell lines were not tested for mycoplasma contamination.

Commonly misidentified lines
(See ICLAC register)

Name any commonly misidentified cell lines used in the study and provide a rationale for their use.

Animals and other research organisms

Policy information about studies involving animals; ARRIVE guidelines recommended for reporting animal research, and Sex and Gender in Research

Laboratory animals

To develop transgenic mice with restricted TdTomato or Sun1 expression, we crossed heterozygous Ntsr1Cre males (B6.FVB(Cg)-Tg(Ntsr1-cre)GN220Gsat/Mmucd, University of California, Davis MMRRC, Mouse Biology Program) to het- or homozygous ROSAlox-stop-lox-TdTomato or ROSAlox-stop-lox-Sun1 lines (B6;129-Gt(ROSA)26Sortm5(CAG-Sun1/sfGFP)Nat/J, Jackson Laboratory, Bar Harbor, Maine). All animals used were between 1 month to 1 year of age.

Wild animals

Provide details on animals observed in or captured in the field; report species and age where possible. Describe how animals were caught and transported and what happened to captive animals after the study (if killed, explain why and describe method; if released, say where and when) OR state that the study did not involve wild animals.

Reporting on sex

Mice of both sexes were used equally, as no sex differences were observed throughout experiments. For instance, we compared the seizure ratings from 4 male and 4 female mice and using the t-test to examine the male/female data we did not find a significant difference at p=0.1955011095).

Field-collected samples

For laboratory work with field-collected samples, describe all relevant parameters such as housing, maintenance, temperature, photoperiod and end-of-experiment protocol OR state that the study did not involve samples collected from the field.

Ethics oversight

All mice were housed in an AALAS-accredited facility on a 14hr light cycle. Husbandry, housing, euthanasia, and experimental guidelines were reviewed and approved by the Institutional Animal Care and Use Committee (IACUC) of Baylor College of Medicine (protocol number: AN-5996).

Note that full information on the approval of the study protocol must also be provided in the manuscript.
